# Metabolomic Discoveries for Traditional Chinese Medicine Efficacy in Alzheimer′s Disease

**DOI:** 10.1155/bmri/2455800

**Published:** 2026-07-31

**Authors:** Huimin Sun, Dongxuan Huang, Fang Yang, Pengfei Zhang, Dexin Yang, Changchun Zeng

**Affiliations:** ^1^ Department of Medical Laboratory, Shenzhen Longhua District Central Hospital, Shenzhen, Guangdong, China, glyy.org; ^2^ Department of Respiratory Medicine, Shenzhen Longhua District Central Hospital, Shenzhen, Guangdong, China, glyy.org; ^3^ Department of Nephrology, Shenzhen Longhua District Central Hospital, Shenzhen Longhua District Key Laboratory for Diagnosis and Treatment of Chronic Kidney Disease, Shenzhen, Guangdong, China, glyy.org

**Keywords:** Alzheimer′s disease (AD), LC-MS, metabolic pathway, metabolomic, traditional Chinese medicine (TCM)

## Abstract

Alzheimer′s disease (AD) is a progressive neurodegenerative disorder characterized by cognitive decline, memory impairment, and behavioral alterations. However, the complex etiology and pathogenesis of AD have thus far precluded the development of satisfactory therapeutic agents. Traditional Chinese medicine (TCM) has garnered increasing recognition for its potential in AD management due to its multicomponent, multitarget therapeutic strategy. Metabolomics, an advanced analytical methodology for investigating metabolic alterations in biological systems, has yielded significant insights into both the therapeutic efficacy and mechanistic underpinnings of TCM interventions for AD. This review synthesizes recent metabolomic findings associated with TCM approaches to AD treatment, identifying key metabolic pathways across diverse biological specimens, including brain tissue, blood, urine, and feces. Through systematic elucidation of these metabolic networks, metabolomics offers substantial potential to facilitate the advancement of TCM‐derived therapeutics for AD, potentially enhancing global patient outcomes.

## 1. Introduction

Alzheimer′s disease (AD) is a progressive neurodegenerative disorder representing the predominant etiology of global dementia cases [1–3]. Clinical manifestations progress from initial cognitive deterioration and episodic memory impairment to severe behavioral alterations, culminating in complete functional dependence [4, 5]. Epidemiological studies reveal a marked escalation in AD prevalence paralleling global demographic aging, posing substantial societal and individual burdens [6, 7]. Current World Health Organization (WHO) estimates indicate approximately 55 million dementia cases worldwide (2020), with projections suggesting this figure may reach 139 million by 2050 [8]. The disease progression involves multifactorial pathophysiological cascades, including but not limited to amyloid‐*β* (A*β*) plaque deposition, neurofibrillary tangles from hyperphosphorylated tau proteins, chronic neuroinflammation, redox imbalance‐induced oxidative stress, and mitochondrial bioenergetic deficits [5, 9, 10]. Despite decades of research, therapeutic development remains hindered by incomplete understanding of disease pathogenesis, extended preclinical latency periods, and limitations in current diagnostic paradigms [11–13]. Emerging evidence demonstrates that traditional Chinese medicine (TCM) interventions for AD management show multidimensional therapeutic effects, demonstrating therapeutic potential in enhancing cognitive performance, ameliorating behavioral pathologies, attenuating pathological progression, and preventing or delaying disease manifestation [14–16].

TCM is a holistic medical system with a history spanning thousands of years. It believes that the human body is a whole in terms of structure and function and has a mutual influence on pathology. TCM therapy plays a synergistic and integrative pharmacodynamic effect of multichannels and multitargets through its multiple components, thus exhibiting unique therapeutic advantages in chronic and multigene complex diseases [17–19]. First, its multicomponent formulations employ synergistic interactions among active ingredients to target complex diseases holistically [20, 21]. Second, TCM therapies are typically associated with fewer adverse effects, owing to their natural origins and harmonized compositions that prioritize balance [19]. It is reported that TCM may play a role in improving cognitive function; inhibiting A*β*/tau pathology, being anti‐inflammatory and antioxidant; and regulating neurotransmitters in AD [22–25]. In addition, TCM can also selectively reshape gut microbial ecosystems, subsequently regulating key microbial metabolites (e.g., short‐chain fatty acids and bile acids) that modulate neuroinflammation implicated in Alzheimer′s pathogenesis [26–28]. Consequently, TCM emerges as a promising platform for developing novel, multitarget therapeutic strategies against AD.

Metabolomics plays a crucial role in AD research by providing a dynamic snapshot of biochemical changes in the body. Its applications include early detection, elucidation of disease mechanisms, drug discovery, and evaluation of therapeutic interventions—particularly TCM [29, 30]. Metabolomics is a powerful tool for decoding TCM, as its systems‐level view aligns with TCM′s holistic philosophy. Like a single TCM formula contains dozens or hundreds of compounds, making it difficult to identify active components and their synergy using traditional methods [31, 32]. Metabolomics addresses this by observing changes in endogenous metabolites after treatment, revealing the pharmacological network through which multiple components work together to restore balance [33, 34]. It shows how the perturbed metabolic profile of a disease model shifts back toward a healthy state after TCM intervention, providing evidence of a rebalancing effect. In summary, metabolomics serves as a molecular bridge that translates TCM principles into measurable metabolites, enzymes, and pathways, thereby enhancing its credibility and facilitating integration with conventional medicine [35–37]. In this review, we have exemplified the study of TCM in animals or clinics on AD and revealed that TCM treatment has significantly changed the metabolic disorders associated with AD, promoting metabolic network reorganization through the restoration of key metabolites and metabolic pathways, which may be the main mechanism basis of TCM′s treatment of AD.

## 2. Search Strategies

A systematic literature search was conducted to identify relevant studies on metabolomic applications in AD research within the framework of TCM. The following search terms and their combinations were defined: “Alzheimer′s disease” (or “AD”), “metabolomics,” and “Traditional Chinese Medicine” (or “TCM”). A comprehensive electronic search was performed across three major databases: PubMed, Google Scholar, and the China National Knowledge Infrastructure (CNKI). No restrictions were placed on publication date or language, although the search primarily focused on English and Chinese literature. The search scope encompassed both animal experiments and clinical studies that employed metabolomic approaches. In addition, the reference lists of relevant reviews and included articles were manually screened to identify further eligible studies.

## 3. Metabolomic Approaches in AD With TCM Interventions

**Figure 1:**
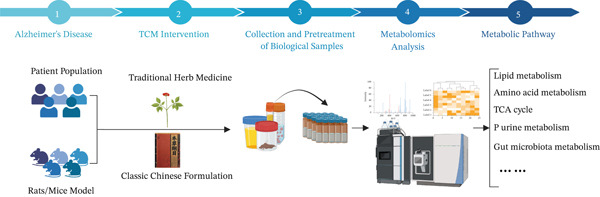
Workflow of metabolomics on TCM for AD.

Metabolomics focuses on the comprehensive analysis of small‐molecule metabolites within biological systems and aims to capture the dynamic changes in metabolic pathways in response to genetic, environmental, or therapeutic interventions [38]. This omics approach combines advanced analytical chemistry with bioinformatics to characterize metabolic signatures in diverse biospecimens, including brain tissue, blood, urine, and feces. In this review, in vivo mouse/rat research and clinical studies on the improvement of AD with TCM will be elucidated, and the relevant main metabolic mechanism will also be mentioned in detail. As illustrated in Figure 1, metabolomic analyses of various tissue samples from AD models treated with traditional herbal medicines or classic Chinese formulas have enabled the comparison of metabolic profiles between AD model groups and TCM‐treated groups, revealing that metabolomics captures how herbal interventions influence AD progression by identifying changes in specific metabolites and simultaneously assessing TCM effects on multiple critical metabolic pathways, including lipid metabolism, amino acid metabolism, the TCA cycle, purine metabolism, and gut microbiota metabolism. From the Table 1 perspective, liquid chromatography–mass spectrometry (LC‐MS) is the main advanced analytical platform in TCM for treating AD studies. It is widely used to analyze metabolites in biological fluids and tissues to elucidate the mechanisms underlying disease and treatment effects, owing to its high sensitivity, separation, and selectivity [72, 73]. It has emerged as a powerful tool for understanding complex diseases, including AD, and for exploring the mechanisms and efficacy of TCM in treating such conditions.

**Table 1:** Application of metabolomics on TCM for AD.

TCM/extracts of TCM/compound	Objects	Biospecimen	Analysis platform	Differential metabolites	Related metabolic pathway	Ref.
Dihuang Yinzi	Mice	Brain	UPLC‐MS	Nicotinic acid, N‐formyl‐L‐glutamic acid, 5‐(2‐hydroxyethyl)‐4‐methylthiazole, D‐gulono‐1,4‐lactone, norepinephrine, 3‐methylotrophicacid, and palmitic acid	Nicotinite and nicotinamide metabolism, pertussis, cAMP signaling pathway, and cysteine and methionine metabolism	Zhang et al. [39]
*Schisandra chinensis* (Turcz.) Baill–*Acorus tatarinowii* Schott	Rats	Brain	UPLC‐MS/MS	PC(20:1(11Z)/16:1(9Z)), stearoyl sphingomyelin, PC(14:0/22:4(7Z,10Z,13Z,16Z)), ceramide (d18:1/18:0), PC(16:0/16:0), conivaptan, quinaprilat, 1‐oleoylglycerophosphoinositol, N‐undecylbenzenesulfonic acid, 2‐dodecylbenzenesulfonic acid, LysoPE(20:4(8Z,11Z,14Z,17Z)/0:0), linoleic acid, mesterolone, DG(18:1(9Z)/18:2(9Z,12Z)/0:0), inosine, elaidic acid, docosahexaenoic acid, and adrenic acid	Linoleic acid metabolism, biosynthesis of unsaturated fatty acids, glycerophospholipid metabolism, and alpha‐linolenic acid metabolism	Hao et al. [40]
Ginsenosides Rg1 and Rg2	Mice	Brain	UPLC/MS	Hypoxanthine, dihydrosphingosine, hexadecasphinganine, LPC C 16:0, LPC C 18:0, phytosphingosine, LPC C 13:0, LPC C 15:0, LPC C 18:1, and LPC C 18:3	Lysophosphatidylcholines (LPCs), hypoxanthine, and sphingolipids metabolism	Li et al. [41]
Radix ginseng and *Schisandra chinensis*	Mice	Brain	AFADESI‐MSI	28 metabolites	Arginine and proline metabolism, aspartate and glutamate metabolism, taurine and hypotaurine metabolism, TCA cycle, glycine, serine, and threonine metabolism, purine metabolism, glutathione metabolism, and fatty acid metabolism	Fan et al. [42]
Bushen Tiansui formula	Rats	Hippocampus cerebral cortex	LC‐MS	111 metabolites (hippocampus)	D‐Glutamine and D‐glutamate metabolism (hippocampus) Cysteine and methionine metabolism (cerebral cortex)	Li et al. [43]
				105 metabolites (cerebral cortex)		
Bushen Tiansui formula	Rats	Cerebral cortex	UPLC‐MS	PE(15:0/14:1(9Z)), Cer(d18:0/22:0), fasciculic acid B, citbismine A, and 4‐nitrophenol	Sphingolipid metabolism; glycerophospholipid metabolism; alanine, aspartate, and glutamate metabolism; and D‐glutamine and D‐glutamate metabolism	Yi et al. [44]
Kai‐Xin‐San	Mice	Brain	UPLC‐Q‐TOF‐MS	2‐Tridecenal, stearic acid, 2‐oxo‐heneicosanoic acid, 9‐deoxy‐9‐methylene‐16, and 16‐dimethyl‐PGE2, 22‐deoxy‐20,21‐dihydroxyecdysone, and PS(21:0/0:0)	Glycerophosphate metabolism, fatty acid biosynthesis, and unsaturated fatty acid biosynthesis	Zhang et al. [45]
Kai‐Xin‐San	Rats	Brain	UPLC/ESI‐Q‐TOF/HDMS	36 metabolites	Linoleic acid metabolism, arachidonic acid metabolism, glycerophospholipid metabolism, sphingolipid metabolism, primary bile acid biosynthesis, secondary bile acid biosynthesis, taurine and hypotaurine metabolism, tyrosine metabolism, lysine degradation, histidine metabolism, pyrimidine metabolism, and purine metabolism	Chu et al. [22]
		Serum				
*Alpinia oxyphylla* fructus	Rats	Brain	UHPLC‐MS/MS	9 metabolites (brain)	Amino acid metabolism, lipid metabolism, and energy metabolism	Sun et al. [46]
		Plasma		23 metabolites (plasma)		
Polygonati rhizoma	Mice	Brain	UPLC‐HRMS	/	Cell growth and death, amino acid metabolism, nervous system, and glycan biosynthesis and metabolism	Xiaojuan et al. [47]
		Blood				
*Xanthoceras sorbifolium* Bunge husks	Rats	Brain	UHPLC‐Q‐TOF‐MS/MS	19 metabolites (brain)	Linoleic acid metabolism; arachidonic acid metabolism; alanine, aspartate, and glutamate metabolism; D‐glutamine and D‐glutamate metabolism; sphingolipid metabolism; and phenylalanine metabolism	Rong et al. [48]
		Serum				
				12 metabolites (serum)		
Danggui‐Shaoyao‐San	Mice	Serum	LC‐MS	Upregulation: L‐cysteine, uracil, argininosuccinic acid, citric acid, and sedoheptulose‐7‐phosphate	Energy metabolism	Wu et al. [49]
				Downregulation: serine, L‐alanine, 3‐phenyllactic‐acid		
Wine‐processed *Schisandra chinensis* polysaccharide	Mice	Serum	LC‐MS/MS	Upregulation: Seryllysine	Ala, Asp, and Glu metabolism and the TCA cycle	Wu et al. [50]
				Downregulation: 42 metabolites		
*Rhodiola crenulata* extract	Rats	Serum	FT‐ICR MS	FA (18:1), FA (18:2), FA (18:3), Cer (d18:0/20:0), Cer (d18:1/24:0), LPC (O‐18:0), LPC (P‐18:0), PC (18:2/18:0), PC (18:2/18:2), PC (18:2/20:4), PC (35:1), PC (35:4), PC (36:1), PC (O‐32:0), SM (d18:0/16:1), and SM (d40:2)	Linoleic acid metabolism, *α*‐linoleic acid metabolism, sphingolipid metabolism, and ether lipid metabolism	Sun et al. [51]
*Rhodiola crenulata* extract	Rats	Serum	HPLC‐FT‐ICR‐MS	Sphinganine, LysoPC (15:0), LysoPC (16:0), LysoPC (16:1(9Z)), LysoPC (17:0), LysoPC (18:0), LysoPC (18:1), LysoPC (18:3), LysoPC (18:2(9Z,12Z)), LysoPC (20:2(11Z,14Z)), LysoPC (20:3), LysoPC (20:4), l‐tryptophan, LysoPC (22:6(4Z,7Z,10Z,13Z,16Z,19Z)), propionylcarnitine, butyrylcarnitine, and linoleyl carnitine	Tryptophan metabolism, sphingolipid metabolism, and glycerophospholipid metabolism	Zhang et al. [52]
Bushen Tiansui formula	Rats	Serum	LC‐MS	Upregulation: 38 metabolites	Linoleic acid metabolism, *α*‐linolenic acid metabolism, glycerophospholipid metabolism, tryptophan metabolism, and arginine and proline metabolism	Zhang et al. [34]
				Downregulation: 40 metabolites		
*Schisandra chinensis*	Rats	Serum	UPLC‐TOF‐MS	Leukotriene C4, leukotriene E4, mesobilirubinogen, leukotriene A4, arachidonic acid, retinyl ester, *α*‐linolenic acid, linoleic acid, inositol 1,3,4,5‐tetraphosphate, citric acid, oxoglutaric acid, bilirubin, 11‐*cis*‐retinol, and retinoyl *β*‐glucuronide	Linoleic acid metabolism; *α*‐linoleic acid metabolism; arachidonic acid metabolism; biosynthesis of unsaturated fatty acids; retinol metabolism; valine, leucine, and isoleucine biosynthesis; sphingolipid metabolism; the TCA cycle; and tryptophan metabolism	Yang et al. [16]
*Ginkgo biloba* L. leaf extract	Mice	Plasma	LC‐MS/MS	Oleoylethanolamide, palmitic amide, brassylic acid, 5 ^′^‐methylthioadenosine, ketosphingosine, and LysoPC(17:0)	Histidine metabolism; alanine, aspartate, and glutamate metabolism; glycerophospholipid metabolism; glycerolipid metabolism; and pyruvate metabolism	Liu et al. [53]
Breviscapine	Mice	Plasma	HPLC‐Q‐TOF‐MS	Indoleacrylic acid, C16 sphinganine, LPE (22:6), sulfolithocholic acid, LPC (16:0), PA (22:1/0:0), taurodeoxycholic acid, and PC (0:0/18:0)	Tryptophan metabolism, phospholipid metabolism, and cholesterol metabolism	Xia et al. [54]
*Alpiniae oxyphyllae* fructus	Mice	Plasma	UHPLC‐MS/MS	Sphinganine (Sa), LysoPE (20:2), glycocholic acid (GCA), and deoxycholic acid (DCA)	Sphingolipid metabolism, TCA cycle, glycerophospholipid metabolism, tyrosine metabolism, and primary bile acid biosynthesis	Zhou et al. [55]
*Callicarpa kwangtungensis* Chun	Mice	Plasma	UPLC‐QE Plus‐MS/MS	/	Phenylalanine metabolism and the TCA cycle	Liu et al. [56])
Total ginsenosides	Mice	Plasma	UHPLC–TOF‐MS	Proline, valine, tryptophan, LPC (14:0), LPC (15:0), LPC (15:1), LPC (17:0), LPC (18:2), LPC (18:3), LPC (20:4), acetylcarnitine, palmitoylcarnitine, vaccenylcarnitine, phytosphingosine, N‐eicosanoylethanolamine, hexadecenoic acid, docosahexaenoic acid, docosapentaenoic acid, and octadecadienoic acid	Amino acid pathway, including proline, valine, and tryptophan; carnitine metabolism, including acetylcarnitine, acylcarnitines, and polyunsaturated fatty acids; and sphingolipid metabolism, including phytosphingosine	Gong et al. [57]
Ginsenosides Rg1 and Rb1	Mice	Plasma	UPLC‐MS	Lysophosphatidylcholine (LPC), tryptophan, phenylalanine, and dihydrosphingosine	Lecithin, amino acid, and/or sphingolipid metabolism	Li et al. [58]
Huanglian Jiedu decoction in combination with donepezil	Patients	Urine	LC–MS	1537 metabolites (107 upregulated and 1430 downregulated) (urine)	Lipid metabolism and glutamic acid metabolism	Xu et al. [25]
		Serum				
				351 metabolites (206 upregulated and 145 downregulated) (serum)		
*Schisandra*–*Evodia* herb pair	Rats	Serum	UPLC‐Q‐TOF‐MS	40 metabolites (serum)	Taurine and hypotaurine metabolism, linoleic acid metabolism, *α*‐linolenic acid metabolism, glycerophospholipid metabolism, and arachidonic acid metabolism	Pang et al. [59]
		Urine		38 metabolites (urine)		
Lignans from *Schisandra chinensis* (Turcz.) Baill	Rats	Plasma	UPLC‐Q‐TOF‐MS	17 metabolites (plasma)	Amino acid metabolism, vitamin metabolism, and biosynthesis of unsaturated fatty acids	Zhou et al. [60]
		Urine		36 metabolites (urine)		
*Schisandra chinensis* polysaccharides	Rats	Serum	UPLC‐Q‐TOF‐MS	21 metabolites (serum)	Linoleic acid metabolism, alpha‐linolenic acid metabolism, and arachidonic acid metabolism	Fu et al. [61]
		Urine		19 metabolites (urine)		
*Schisandra chinensis* (Turcz.) Baill (*S. chinensis*)	Rats	Urine	UPLC‐MS	Upregulated: *γ*‐Aminobutyric acid (GABA), 5‐hydroxytryptamine (5‐HT), acetylcholine (Ach), norepinephrine (NE), and glycine (Gly)	Polyunsaturated fatty acid metabolism and amino acid and vitamin metabolism	Wei et al. [24]
		Plasma				
				Downregulate: aspartic acid (Asp)		
Gan Mai Da Zao decoction	Rats	Plasma	UPLC‐Q‐TOF‐MS	2‐hydroxyestradiol, biotin, 4,6‐dihydroxyquinoline, morphine‐6‐glucuronide, and riboflavin	Steroid hormone biosynthesis, biotin metabolism, tryptophan metabolism, drug metabolism‐cytochrome P450, and riboflavin metabolism	Cui et al. [62]
		Urine				
Hengqing II prescription	Patients	Urine	UHPLC‐MS	Upregulation: 16 metabolites	Linoleic acid metabolism; arginine biosynthesis; alanine, aspartate, and glutamate metabolism; D‐glutamine and D‐glutamate metabolism; and glycine, serine, and threonine metabolism	Meng et al. [63]
				Downregulation: 50 metabolites		
*Schisandra* polysaccharide	Rats	Urine	UHPLC‐Q‐TOF‐MS	Upregulation: 23 metabolites	Tryptophan, tyrosine, phenylalanine, taurine and hypotaurine, arginine and proline, purine and pyrimidine, nicotinate and nicotinamide, and lysine	Liu et al. [23]
				Downregulation: 15 metabolites		
Radix ginseng–*Schisandra chinensis* herb pair	Rats	Urine	UHPLC‐Q‐TOF‐MS	3‐Indole carboxylic acid glucuronide, 3‐methyldioxyindole, 4,6‐dihydroxyquinoline, hippuric acid, phenylacetylglycine, 5‐L‐glutamyl‐taurine, pantothenic acid, creatinine, 4‐guanidinobutanoic acid, 4‐pyridoxic acid, indoxyl sulfate, *p*‐cresol glucuronide, *p*‐cresol sulfate, 2‐phenylethanol glucuronide, uric acid, and citric acid	Phenylalanine and tyrosine metabolism, tryptophan metabolism, and purine metabolism	Wang et al. [64]
Shengmai San	Mice	Urine	UPLC‐Q‐TOF‐MS	34 metabolites	Nicotinate and nicotinamide metabolism, tryptophan metabolism, catecholamine biosynthesis, vitamin B6 metabolism, phenylalanine and tyrosine metabolism, oxidation of branched‐chain fatty acids, beta oxidation of very long chain fatty acids, tyrosine metabolism, protein biosynthesis, purine metabolism, and TCA cycle	Zhang et al. [65]
Dihuang Yinzi	Mice	Urine	UPLC‐HRMS	*Cis*‐aconitic acid, citric acid, phosphoenolpyruvic acid, pyruvic acid, fumaric acid, succinic acid, L‐lactic acid, malonic acid, nicotinamide, guanine, hypoxanthine, nicotinic acid, glycerol‐3‐phosphate, and choline	Glycerophospholipid metabolism, nicotinate/nicotinamide metabolism, glycolysis, and the TCA cycle	Han et al. [66]
*Corallodiscus flabellatus* (Craib) B. L. Burtt	Mice	Urine	UPLC‐Q‐TOF‐MS	Upregulation: 7 metabolites	Glycine, serine, and threonine metabolism; arginine and proline metabolism; and phenylalanine metabolism	Wang et al. [67]
				Downregulation: 21 metabolites		
Ding‐Zhi‐Xiao‐Wan	Rats	Urine	UHPLC‐Q‐TOF‐MS	Upregulation: 14 metabolites	Taurine and hypotaurine metabolism, tryptophan metabolism, and phenylalanine metabolism	He et al. [68]
				Downregulation: 12 metabolites		
*Schisandra chinensis* polysaccharide	Rats	Feces	UPLC‐Q‐TOF‐MS	Upregulation: 13‐HOTE, deoxycholic acid, eicosapentaenoic acid, oxoglutaric acid, succinic acid, docosapentaenoic acid, alpha‐linolenic acid, linoleic acid, and 24‐hydroxycholesterol	Linoleic acid metabolism, arachidonic acid metabolism, *α*‐linolenic acid, and the TCA cycle	Fu et al. [26]
				Downregulation: 4‐(2‐Aminophenyl)‐2,4‐dioxobutanoic acid, arachidonic acid, 8,11,14‐eicosenoic acid, adrenic acid, chenodeoxycholic acid, 7‐ketodeoxycholic acid, cholic acid, 13S‐hydroxyoctadecadienoic acid, prostaglandin E2, and docosahexaenoic acid		
*Schisandra chinensis*–*Acorus tatarinowii* Schott	Rats	Feces	UPLC‐Q‐TOF‐MS	Phytosphingosine, sphinganine, LysoPC(18:2(9Z,12Z)), cortexolone, linoleic acid, LysoPC(O‐18:0), androsterone, LysoPC(P‐18:0), oleic acid, 3a,7a,12a‐trihydroxy‐5bcholestan‐26‐al, 17a,20a‐dihydroxycholesterol, PC(18:0/18:2(9Z,12Z)), PC(16:1(9Z)/P‐18:1(11Z)), riboflavin, lithocholate 3‐oglucuronide, D‐urobilinogen, LysoPC(20:2(11Z,14Z)), and 21‐deoxycortisol	Linoleic acid metabolism, steroid hormone biosynthesis, sphingomyelin metabolism, and riboflavin metabolism	Shan et al. [28]
Radix ginseng–*Schisandra chinensis* herb pair	Rats	Feces	UPLC‐LTQ‐Orbitrap‐MS	16 metabolites	Bile acid biosynthesis, sphingolipid metabolism, porphyrin, and chlorophyll metabolism	Wang et al. [69]
Schisanlactone E	Mice	Brain	LC‐MS	Isosakuranetin, 5‐KETE, 4‐methylcatechol, and sphinganine	Carbohydrate metabolism; neuroactive ligand–receptor interactions; and alanine, aspartate, and glutamate metabolism	Song et al. [70]
		Fecal				
Icariin	Mice	Fecal	LC–MS	Fecal metabolomics: 687 differential metabolites (265 upregulated and 422 downregulated)	Sphingolipid metabolism	Liu et al. [71]
		Serum				
				Serum metabolome: 111 differential metabolites (40 upregulated and 61 downregulated)		

## 4. Metabolomic Analysis in AD With TCM Interventions

Recently, with the application and development of metabolomics in AD, accumulated research has reported the application of TCM in the study of AD. In this review, metabolomic studies in AD were classified according to the TCM or compound prescriptions used, biological samples, metabolic analysis platforms, differential metabolites, and metabolic pathways. The results are shown in Table 1. In the study of treating AD with TCM, metabolomics can be applied to various biological samples, including brain tissue, blood, urine, and feces. Each sample type contains unique metabolomic information and is mainly involved in different metabolic pathways [74]. Those are discussed in some detail below.

### 4.1. Brain‐Based Metabolomics in AD With TCM Interventions

Brain tissue metabolomics offers a direct assessment of metabolic perturbations in AD, capturing disease‐specific pathological hallmarks, including A*β* deposition, tau hyperphosphorylation, and neuroinflammatory responses [75, 76]. Unlike peripheral samples (e.g., blood or urine), brain tissue metabolomics avoids confounding factors from systemic metabolism, providing more specific insights into central nervous system (CNS)–related changes. Over the past decade, researchers have employed brain metabolomics to study the effects of TCM on AD, including the Bushen Tiansui formula (BSTSF), Ginsenosides Rg1 and Rg2 (G‐Rg1 and G‐Rg2), Kai‐Xin‐San (KXS), Radix ginseng and *Schisandra chinensis* (GS), Polygonati rhizoma (PR), *Schisandra chinensis* (Turcz.) Baill–*Acorus tatarinowii* Schott (Sc‐At), and Dihuang Yinzi (DHYZ) [22, 39–45, 77]. These studies elucidate how TCM modulates critical brain metabolic pathways in AD models, primarily targeting lipid metabolism, amino acid metabolism, and purine metabolism.

The study reported that the brain of the AD rats has a distinct lipidomic profile, including dysregulated sphingolipid metabolism, glycerophospholipid metabolism, linoleic acid (LA) metabolism, and arachidonic acid (AA) metabolism [44]. The study identified that in the brain, imbalances in the contents of various classes of sphingolipids (including dihydrosphingosine, hexadecasphinganine, and phytosphingosine) can result in neuronal dysfunction and apoptosis of brain cells, ultimately leading to AD [41, 78–80]. Li et al.′s mechanistic investigation elucidated that disorders of sphingolipid levels are also observed in the brains of AD mice and indicated that G‐Rg1 treatment upregulated all three sphingolipid species, whereas G‐Rg2 treatment just increased the levels of dihydrosphingosine and hexadecasphinganine [41]. The result was that the impaired cognitive function and increased hippocampal Ab deposition in AD mice were ameliorated by G‐Rg1 and G‐Rg2. Glycerophospholipid levels correlate with amyloid and neurofibrillary pathology severity in AD [81]. Since sphingolipids and glycerophospholipids are part of phospholipid metabolism, their dysregulation is closely linked to apolipoprotein E4 (APOE4), a key determinant of brain phospholipid homeostasis [43]. The phospholipid dysregulation contributes to APOE4‐associated cognitive deficits in AD pathogenesis, and it could exacerbate the intraneuronal accumulation of A*β* and plaque deposition in the brain parenchyma [82, 83]. Some studies suggest that BSTSF treatment could restore some dysregulated metabolites and abnormal lipid metabolism in the cerebral cortex of AD rats, which might treat AD through regulating the APOE4 and phospholipid metabolism [43, 44]. The other study found that the content of 22‐deoxyy‐20,21‐dihydroyecdysone (a glycerol phosphate lipid molecule) was significantly increased in the brain tissue of mice, indicating that the metabolic abnormality of glycerophosphate is closely related to the deposition of A*β* [45]. However, after oral administration of KXS, the levels of 22‐deoxyy‐20,21‐dihydroyecdysone in mice were downregulated and can reduce the deposition of A*β* protein [45]. In addition, studies have reported disturbances in LA and AA metabolism in the brains of AD model rats. LA was found to inhibit A*β*_1-42_ cytotoxicity; however, excessive LA stimulation was also reported to promote tau protein and A*β* assembly in neurons [84, 85]. AA has been shown to influence both tau protein and A*β* aggregation in neurons while also acting as a neuroinflammatory agonist—a potential contributor to AD pathogenesis [86, 87]. Notably, Rong et al. identified that *Xanthoceras sorbifolium* husk extract (XSE) confers neuroprotection through normalization of LA and AA in hippocampal tissues [48].

Research has demonstrated that glutamine and glutamate metabolism in the brain is intrinsically linked to AD pathogenesis. Clinical investigations have demonstrated that glutamine and glutamate concentrations show a significant reduction in bilateral hippocampal regions of AD patients compared to healthy controls [88]. As a key component of the glutamate metabolic pathway, glutamine is synthesized from glutamate through astrocytic activity [89]. Glutamate, the principal excitatory neurotransmitter, undergoes neuronal decarboxylation through the action of glutamate decarboxylase to produce gamma‐aminobutyric acid (GABA), the primary inhibitory neurotransmitter [90, 91]. Reduced cerebral glutamate concentrations may lead to dysregulation of GABAergic signaling, potentially contributing to the pathogenesis of neurodegenerative disorders, including AD [92]. Li et al. demonstrated that BSTSF ameliorates AD pathology through modulation of glutamine–glutamate metabolic cycling [43]. Furthermore, Rong et al. proposed that XSE preserves metabolic homeostasis in amino acid pathways by maintaining glutamate metabolic equilibrium [48]. This neurotransmitter imbalance may represent a critical pathway in AD progression, warranting further investigation into metabolic interventions.

Purine metabolism in the brain is also intricately associated with AD pathogenesis [93]. As a core intermediate in purine metabolism, hypoxanthine serves as a dynamic indicator of pathway equilibrium. Experimental studies have demonstrated that hypoxanthine enhanced acetylcholinesterase (AChE) activity in the hippocampus and striatum of 15‐ and 30‐day‐old mice when added to the incubation medium [94]. Notably, AChE activity is mechanistically involved in AD pathophysiology through its role in cholinergic neurotransmission disruption. Furthermore, hypoxanthine has been shown to inhibit Na^+^, K^+^‐ATPase activity while inducing oxidative stress in the rat striatal region [41]. Such oxidative stress constitutes a primary pathogenic mechanism in AD and has been found to impair the brain memory of mice [95]. Experimental evidence suggests that G‐Rg1 and G‐Rg2 modulate purine metabolism through xanthine oxidase inhibition, effectively regulating its endogenous concentration and subsequently ameliorating AChE hyperactivity and oxidative damage, thereby mitigating AD pathological features [41]. These findings highlight the potential of purinergic pathway modulation as a therapeutic strategy against AD progression.

### 4.2. Blood‐Based Metabolomics in AD With TCM Interventions

Blood serves as a rich repository of diverse metabolites that continuously mirror an organism′s physiological and pathological states in real‐time, rendering it the most widely utilized sample in metabolomic studies [96, 97]. Blood metabolomics provides a comprehensive framework for understanding systemic metabolic dysregulation, encompassing both CNS and peripheral perturbations, thereby offering unique insights into interorgan communication between the brain and visceral systems (e.g., hepatic, renal, and gastrointestinal systems) [98, 99]. Recent advances have employed blood metabolomics to evaluate TCM interventions for AD, including Danggui‐Shaoyao‐San (DSS), *Rhodiola crenulata* extract (RCE), BSTSF, *Schisandra chinensis* (SCS), *Ginkgo biloba* L. leaf extract (GBLE), breviscapine, *Alpiniae oxyphyllae* fructus (AOF), *Callicarpa kwangtungensis* Chun (CK), and ginsenosides [16, 34, 49, 51, 53–57]. Mechanistic studies demonstrate TCM‐mediated regulation of hematogenous metabolic networks in AD models, principally involving lipid metabolism, amino acid metabolism, and the TCA cycle.

Emerging evidence from metabolomic studies reveals a strong association between abnormal blood lipid metabolism and AD, particularly involving dysregulation in sphingolipid metabolism, LA metabolism, *α*‐linolenic acid (ALA) metabolism, AA metabolism, and polyunsaturated fatty acid (PUFA) metabolism. Notably, the most pronounced alterations occur in sphingolipid metabolism in serum/plasma samples from AD mouse/rat models [52, 53]. Disruptions in sphingolipid metabolism may contribute to AD‐related neuropathology by altering A*β* precursor protein processing, impairing synaptic function, and triggering neuronal death [78]. Among them, perturbations in ceramides, which are the central molecule of sphingolipid biosynthesis, were detrimentally associated with several pathological aspects of AD [100]. Clinical data indicate elevated ceramide levels are present in AD patients with mild‐to‐moderate symptoms, while elevated plasma ceramide levels in elderly females correlate with increased AD risk [79, 80, 101]. Similarly, Sun et al. documented significantly elevated ceramide concentrations in AD rat serum, with RCE treatment normalizing these levels [51]. This suggests that RCE exerts neuroprotective effects through ceramide level reduction. Biochemical analyses reveal that sphinganine undergoes conversion to ceramide, and elevated ceramide levels accelerate interleukin‐2 (IL‐2) and interleukin‐6 (IL‐6) production, exacerbating neuroinflammation [78]. High‐dose RCE administration elevates sphinganine concentrations to control‐equivalent levels. Zhang et al. further corroborate that RCE′s therapeutic mechanisms involve sphingolipid metabolism modulation [52]. In sphingolipid pathways, sphingosine generated through ceramide deacylation demonstrates neuroprotective properties, while serum sulfatide levels independently correlate with atherosclerotic progression by promoting vascular inflammatory responses [102, 103]. Chu et al. observed decreased serum sphingosine levels and elevated sulfatide concentrations in AD rats, indicating impaired sphingolipid metabolism ameliorated by KXS through pathway regulation [22]. Sphingomyelins (SMs), synthesized from phosphatidylcholines (PCs) and ceramides via SM synthase catalysis, play crucial roles in neuronal signaling pathways. Current research establishes that SM metabolism perturbations contribute to the AD pathophysiology [104, 105]. Liu et al. identified elevated plasma concentrations of SM (d16: 0/27:5), SM (d16:0/24:2), and SM (d16:0/26:5) in AD mice [53]. Sun et al. reported a marked elevation of SMs in AD rat serum, with subsequent normalization post‐RCE administration, indicating that SM reduction mediates RCE′s therapeutic effects [51]. In addition, the LA, ALA, and AA metabolic pathways play pivotal roles in AD pathogenesis, functioning as key regulatory molecules in disease progression. Evidence indicates that LA is enzymatically converted to 9‐*cis*, 11‐*trans*‐octadecadienoate (a conjugated LA derivative) via linoleate isomerase activity, exerting neuroprotective effects against neurotoxic agents and reducing tau hyperphosphorylation [16, 106]. Serum ALA concentrations demonstrate an inverse correlation with dementia risk, while AA metabolites are recognized contributors to inflammatory processes underlying A*β*‐induced pathogenesis [107–109]. Current research utilizing serum metabolomics has revealed that SCS administration induces partial restoration of metabolic disturbances in AD rat models through the modulation of LA, ALA, and AA pathways [16, 59, 61]. These findings demonstrate significant improvements in spatial learning and memory functions in SCS‐treated groups. Substantiating these observations, Sun et al. documented elevated AA levels accompanied by decreased LA and ALA concentrations in AD rat serum, all of which were normalized following RCE treatment, confirming its disease‐modifying potential through metabolic pathway regulation [51]. As was reported, a disorder of PUFA metabolism is also one of the key factors leading to AD. In PUFA biosynthesis, docosahexaenoic acid (DHA), as a representative PUFA, not only reduces vascular A*β* deposition and overall A*β* burden but also enhances synaptic and neurotransmitter function, improving learning and memory abilities, and its serum concentration in AD was significantly lower [110–113]. However, treatment with TCM (SCS and total ginsenosides) demonstrates therapeutic potential in AD models by restoring dysregulated PUFA metabolism [16, 57].

According to related literature, blood metabolomic studies in AD highlight alterations in tryptophan metabolism, which plays a pivotal role in AD pathogenesis and progression. Tryptophan metabolism primarily occurs through the serotonin and kynurenine pathways. Accumulating evidence demonstrates reduced serotonin levels in AD patients, suggesting that enhancing serotonin signaling could be a potential disease‐modifying therapy [114, 115]. Xia et al.′s research team reported markedly elevated concentrations of indoleacrylic acid (IAA) in the AD model group, showing concentration normalization post breviscapine intervention [54]. As a result, they supposed that breviscapine exerted its neuroprotective effects by interrupting the metabolic pathway of tryptophan to produce IAA and, in consequence, an increase in serotonin levels. In another study, the levels of serotonin also showed an upregulating trend in the AD model rats after treatment with BSTSF [34]. Similarly, SCS treatment induced quantitative restoration of IAA levels in the AD model, suggesting a therapeutic mechanism involving tryptophan metabolic modulation [16]. Kynurenine, a key tryptophan catabolite, exhibits complex dual roles in AD pathogenesis through neurotoxic–neuroprotective equilibrium disruption [116, 117]. Li et al. also found that plasma tryptophan level was reversed by treatment with G‐Rg1/G‐Rb1 [58]. The investigators hypothesize that the increase in tryptophan induced by G‐Rg1 and G‐Rb1 results from the suppression of the kynurenine pathway. These findings collectively highlight the therapeutic potential of tryptophan metabolic pathway modulation in AD management.

The TCA cycle plays a key role in AD pathophysiology, with its dysfunction intricately linked to energy metabolism, oxidative stress, and neuronal death in AD [118, 119]. Significant impairments have been reported in multiple enzymes from the TCA cycle in AD patients, leading to abnormal levels of intermediates of the TCA cycle and related compounds, such as decreased serum concentration of 2‐oxoglutarate (oxoglutaric acid) [16]. Yang et al.′s research team systematically demonstrated that SCS intervention restores *α*‐ketoglutarate homeostasis, suggesting therapeutic effects of SCS might be related to the modulation of the impaired TCA cycle [16]. Another study reported that a reduction in citric acid was observed in the serum, providing biochemical evidence of mitochondrial TCA cycle impairment [120]. Rong et al.′s study revealed that the serum levels of citric acid were elevated in the AD model group after treatment with XSE and showed better protection of energy metabolism in mitochondria [48]. Likewise, Liu et al. suggested that CK may play a role in alleviating metabolic disorders and neuroprotection by improving the TCA cycle [56].

### 4.3. Urine‐Based Metabolomics in AD With TCM Interventions

Urine contains metabolites filtered from the blood, providing insights into systemic metabolic alterations, including those influenced by the gut–brain axis and peripheral organs. Urine samples are ideal sources of biomarkers for clinical analysis, reflecting many metabolic end products and metabolites in the organism due to their easy availability and noninvasive sampling [121, 122]. Although the mechanisms by which urinary metabolites reflect brain alterations need further elucidation, urinary metabolomic studies have revealed metabolite signatures and changes in pathways related to neurological disorders [123]. Over the past decade, researchers have studied some of the TCM used to treat AD by urine, such as *Schisandra* polysaccharide (SCP), GS, Ding‐Zhi‐Xiao‐Wan (DZXW), *Corallodiscus flabellatus* (Craib) B. L. Burtt (CF), DHYZ, Shengmai San (SMS), and Hengqing II Prescription [23, 63–68]. These studies elucidate how TCM modulates critical urine metabolic pathways in AD models, primarily targeting amino acid metabolism, purine metabolism, and gut microbiota metabolism.

Accumulating evidence links urinary amino acid metabolic disturbances, especially in taurine/hypotaurine and arginine/proline metabolisms, to the development of AD pathology. Several studies suggest decreased cerebral taurine concentration in AD patients, with taurine demonstrating inhibitory effects on A*β* aggregation [124]. Experimental evidence indicates that oral taurine administration ameliorates cognitive deficits in APPswe/PS1dE9 transgenic AD mice through direct binding to oligomeric A*β* [23, 125]. Liu et al. and Wang et al. collectively demonstrated decreased urinary concentrations of 5‐L‐glutamyl‐taurine and taurine in AD rats, which showed significant elevation following TCM (SCP and GS) interventions [23, 64]. Additionally, He et al. further observed reduced taurine levels in AD rat models, whereas DZXW treatment restored taurine content, suggesting therapeutic modulation of metabolic pathways [68]. Alterations in the urinary arginine/proline metabolism pathway are increasingly recognized as significant in neurodegenerative disease pathogenesis. Creatinine and 4‐guanidinobutanoic acid, metabolic products of this pathway, demonstrate significant AD‐related alterations. Creatinine serves as a crucial biomarker for renal function assessment, while 4‐guanidinobutanoic acid modulates monocyte and granulocyte activity linked to anti‐inflammatory responses [126]. Elevated urinary levels of creatinine and 4‐guanidinobutanoic acid suggest AD‐induced hyperactivation of arginine/proline metabolism. As an essential precursor for nitric oxide (NO) synthesis, arginine′s metabolic products influence synaptic plasticity through NO signaling in AD [127]. Previous studies indicate that TCM interventions (SCP and GS) significantly modulate creatinine, 4‐guanidinobutanoic acid, and NOS (a key enzyme involved in NO synthesis) levels in AD rats, implying their regulatory effects on AD progression through arginine/proline metabolic regulation [23, 64]. He et al. documented decreased urinary creatinine in AD rats, indicating metabolic pathway dysregulation, while DZXW treatment normalized creatinine levels, further supporting the therapeutic potential of metabolic pathway modulation [68].

Studies have reported that purine metabolites may serve as potential urinary biomarkers, with their dysregulation being closely associated with AD pathogenesis [93, 128, 129]. Uric acid and allantoin represent two critical metabolites in the purine metabolic pathway. Uric acid, the terminal product of purine metabolism, exhibits metabolic dysregulation that may contribute to various pathologies, including gout, cardiovascular disorders, and renal dysfunction [130–134]. Notably, cardiovascular risk factors may not only contribute to cerebrovascular disorders but also directly influence A*β* deposition, thereby impacting cognitive function [135, 136]. Wang et al. demonstrated significant dysregulation of uric acid metabolism in AD models, which was effectively normalized following GS compound administration [64]. Allantoin is excreted as the final product of reactions involving the oxidation of various reactive oxygen species (ROS). Clinical studies have identified elevated allantoin concentrations in AD patients, with these metabolic alterations potentially preceding clinical symptom manifestation [137, 138]. Wang et al. think that CF extract downgraded allantoin levels by purine metabolism and further increased the oxidation in the brain [67]. Another study observed elevated concentrations of both uric acid and allantoin in the AD group [68]. DZXW administration significantly downregulated both metabolites, indicating that DZXW might eliminate oxygen free radicals in the body and alleviate the purine metabolism disorder in AD rats.

The gut microbiota exerts a significant influence on AD pathogenesis through bidirectional communication with the CNS via microbial metabolites and immune–neuroendocrine pathways [139–141]. The report proved that TCM can be absorbed and metabolized by gut microbiota [142]. Specifically, phenylalanine metabolism by gut microbiota yields hippuric acid and *p*‐cresol metabolites. He et al. observed elevated urinary concentrations of these compounds in AD model rats, leading the authors to hypothesize that gut dysbiosis in AD rats may induce aberrant phenylalanine metabolism [68]. Notably, DZXW treatment normalized these metabolic alterations, demonstrating that DZXW has a certain effect on regulating phenylalanine metabolism and gut microbiota activity. Similarly, GS administration restored metabolite levels to baseline, suggesting therapeutic efficacy against AD‐induced enteric metabolic dysregulation [64]. *p*‐Cresol, a microbial metabolite derived from aromatic amino acid metabolism, exists primarily as conjugated forms (*p*‐cresol glucuronide and sulfate) that have been implicated not only in cardiovascular and cerebrovascular pathologies but also in cognitive dysfunction [143–146]. Liu et al. demonstrated that the levels of *p*‐cresol sulfate increased significantly in AD rats and were downregulated after treatment with SCP, suggesting this intervention may effectively reduce cardiovascular risk to inhibit the occurrence of AD [23].

### 4.4. Fecal‐Based Metabolomics in AD With TCM Interventions

Fecal metabolites are the products of complex metabolic activities performed by the gut microbiota and serve as a bridge between the host and the gut microbes. Consequently, a multiomic strategy of microbiome and fecal metabolomics can provide new insights into the microbiota–gut–brain axis. Studies have reported that gut microbiota alterations can disrupt the host′s physiological homeostasis, potentially contributing to disorders such as AD [147–149]. Over the past decade, there have been a small number of studies that have shown some of the TCM used to treat AD by utilizing feces metabolomics, such as SCP, Sc‐At, and GS [26, 28, 69]. These studies elucidate how TCM modulates key AD‐related metabolic pathways, primarily involving gut microbiota metabolism. Fu et al. conducted a study investigating SCP2′s neuroprotective mechanisms through untargeted fecal metabolomics and microbiome analysis in A*β*_25–35_‐induced AD rats [26]. Results revealed that SCP2 intervention significantly increased *Bacteroides*, *Coprococcus*, and *Paraprevotella* abundance, suggesting neuroprotection via gut microbiota remodeling and improved intestinal permeability. Shan et al. demonstrated that Sc‐At treatment reversed AD‐induced gut microbiota dysbiosis and cerebral inflammation while restoring the intestinal microenvironment [28]. Liu et al. further demonstrated that icariin (ICA) ameliorated gut microbiome dysbiosis and metabolic disorders in APP/PS1 mice, identifying associations between *Alistipes*/*Akkermansia* and sphingolipid metabolism as potential therapeutic mechanisms [71]. Future research is expected to increasingly explore fecal metabolomics in AD, potentially revealing critical pathophysiological mechanisms and identifying disease‐modifying therapeutic targets for this neurodegenerative condition.

## 5. Challenges and Future Directions

Metabolomics demonstrates substantial potential for assessing the therapeutic efficacy of TCM for AD. TCM, characterized by its multitarget and holistic regulatory features, exerts therapeutic effects by modulating multiple key metabolic pathways [150–152]. A comprehensive review of metabolomic studies reveals that the primary metabolic pathways regulated by TCM include lipid metabolism, amino acid metabolism, the TCA cycle, purine metabolism, and gut microbiota metabolism. These pathways are interconnected, forming a complex metabolic network. Lipid metabolism is essential for maintaining the structure and function of neuronal membranes and plays a crucial role in the pathogenesis of AD [67, 153]. In AD, significant lipid metabolic abnormalities—including disruptions in sphingolipids, phospholipids, and cholesterol metabolism—contribute to synaptic damage and neuroinflammation [153, 154]. TCM can restore membrane integrity and improve synaptic plasticity by modulating the activity of lipid metabolism‐related enzymes and metabolite levels. Amino acid metabolism directly affects the balance of neurotransmitters by providing the primary precursor molecules for their synthesis—glutamate from glutamine, GABA from glutamate, and serotonin from tryptophan—thereby regulating excitatory and inhibitory signaling in the CNS [155–157]. In AD, the delicate balance between excitatory and inhibitory neurotransmission is often disrupted, manifesting as glutamate‐induced excitotoxicity and impaired inhibitory neurotransmission [158, 159]. By modulating key amino acid metabolic pathways, including those of glutamate and tryptophan, TCM may help restore the excitation/inhibition balance and mitigate neuronal damage. The TCA cycle represents the core of mitochondrial energy metabolism. In AD, impaired mitochondrial function leads to reduced TCA cycle flux and decreased ATP production, resulting in a neuronal energy crisis [160–162]. TCM can ameliorate this energy deficit and delay neuronal dysfunction by enhancing the activity of key TCA cycle enzymes and replenishing intermediate metabolites. Purine metabolism is closely linked to energy metabolism. When ATP is depleted, purine metabolic pathways are compensatorily activated, and metabolites participate in the regulation of neuroinflammation [163–165]. TCM can improve energy status and suppress inflammatory responses by modulating purine metabolism. Gut microbiota metabolism serves as a central component of the microbiota–gut–brain axis. Through signaling molecules such as short‐chain fatty acids and tryptophan metabolites, the gut microbiota influences intestinal barrier integrity, immune system function, and central neuroinflammation [166–169]. TCM can exert indirect neuroprotective effects by reshaping the structure of the gut microbiota and modulating its metabolite profiles. These five metabolic pathways do not function in isolation; on the contrary, they form a tightly integrated regulatory network through shared metabolic substrates, coupled energy carriers, and intersecting signaling molecules. For instance, short‐chain fatty acids derived from gut microbiota can modulate lipid metabolism and TCA cycle function, amino acid metabolism provides substrates for the TCA cycle, the TCA cycle supports ATP production via oxidative phosphorylation, and ATP is directly linked to purine metabolism as it constitutes the core scaffold of purine nucleotides; in turn, purine metabolites participate in lipid peroxidation and inflammatory regulation [118, 128, 170–172]. This network structure determines that metabolic disturbances in AD are systemic and cascading in nature. The unique advantage of TCM in treating AD lies in its multitarget, multipathway, and holistic regulatory characteristics. Through the synergistic actions of multiple bioactive components, TCM can simultaneously modulate the aforementioned metabolic pathways and restore the coupling relationships among them, shifting the perturbed metabolic state of AD models back toward a healthy equilibrium. Metabolomics provides a comprehensive approach to elucidate this network‐level regulatory effect, thereby establishing a crucial foundation for the modern scientific understanding of TCM intervention in AD.

Despite promising findings, several challenges persist in the application of TCM for AD treatment. First, significant heterogeneity across current studies—in animal models (e.g., transgenic strains and intervention time points), TCM preparations (herb sources, extraction methods, and dosages), metabolomic platforms (sample pretreatment and data processing pipelines), and sample types (brain tissue, serum, urine, and feces)—severely restricts the comparability and reproducibility of results [49, 173–175]. Second, the therapeutic efficacy of TCM is often limited by the low bioavailability of many active compounds, which are poorly absorbed by the body [176]. Third, current research relies primarily on animal models, while clinical studies are limited in number and sample size, with inconsistent diagnostic criteria and intervention protocols. Moreover, the complex etiology of AD patients precludes the identification of uniform metabolic alteration patterns or TCM intervention targets. Thus, the generalizability of existing findings to AD patient populations requires further systematic validation.

Consequently, future research should focus on the following aspects to advance TCM treatment for AD. First, a research consensus needs to be promoted based on unified animal models, standardized TCM preparations, and standardized metabolomic workflows. Second, nanotechnology‐based delivery systems, such as nanoparticles and nanoemulsions, can be employed to encapsulate TCM components, thereby enhancing the solubility and absorption of herbal compounds and maximizing their therapeutic potential against AD [176]. Third, clinical translational research should be advanced through large‐scale, prospective clinical studies employing standardized TCM formulations, combined with metabolomic techniques, to establish efficacy prediction models and individualized metabolic biomarker systems for TCM treatment in AD patients. Fourth, metabolomics should be integrated with genomics, transcriptomics, proteomics, and metagenomics to construct a multilevel “gene–protein–metabolite–phenotype” regulatory network for TCM intervention in AD, providing theoretical support for precision diagnosis and treatment [177, 178]. Finally, improved detection equipment and advanced medical sensing technologies for AD monitoring should be developed, alongside personalized medicine approaches to tailor TCM interventions to individual metabolic profiles [179].

## 6. Conclusion

Metabolomics has emerged as a critical tool for elucidating the molecular mechanisms underlying TCM intervention in AD. By systematically characterizing the metabolic profiles of AD models before and after TCM treatment, metabolomic studies have demonstrated that TCM exerts its therapeutic effects through a multitarget, multipathway holistic regulatory approach, coordinately influencing five key metabolic pathways—lipid metabolism, amino acid metabolism, TCA cycle, purine metabolism, and gut microbiota metabolism. This approach provides visualizable molecular evidence for the TCM principles of “holistic concept” and “syndrome differentiation and treatment” while also establishing a new research paradigm for elucidating the multicomponent synergistic actions of TCM. Despite the promising potential of TCM for treating AD, several critical challenges remain, including the complex etiology of AD, poor understanding of metabolic pathway interactions, a lack of well‐designed clinical studies, and low bioavailability of many bioactive TCM compounds. Overcoming these challenges will depend on unified study standards, advanced delivery systems to boost bioavailability, and multiomic integration to construct regulatory networks for precision diagnosis and personalized AD treatment. In summary, metabolomics has laid an important foundation for understanding the systemic regulatory mechanisms of TCM in AD treatment, yet this field remains in its early stages of development. Continued research will facilitate further elucidation of the metabolic network regulatory patterns underlying TCM intervention in AD and provide novel scientific evidence for the precision diagnosis and treatment of this complex systemic disorder.

## Author Contributions

H.S., D.H., and F.Y. wrote the main manuscript text. H.S. prepared the graphical abstract, Figure 1, and Table 1. C.Z. revised the manuscript. All authors reviewed the manuscript. H.S., D.H., and F.Y. contributed equally to this work.

## Funding

This study was funded by the Scientific Research Projects of Medical and Health Institutions of Longhua District, Shenzhen (2023008).

## Conflicts of Interest

The authors declare no conflicts of interest.

## Data Availability

Data sharing is not applicable to this article as no datasets were generated or analyzed during the current study.
